# Perceptions toward antiretroviral therapy and delayed ART initiation among people living with HIV in Changsha, China: mediating effects of treatment willingness

**DOI:** 10.3389/fpubh.2023.1105208

**Published:** 2023-06-13

**Authors:** Yaqin Zhou, Yixuan Li, Xueling Xiao, Han-Zhu Qian, Honghong Wang

**Affiliations:** ^1^Xiangya School of Nursing, Central South University, Changsha, China; ^2^School of Public Health, Yale University, New Haven, CT, United States

**Keywords:** human immunodeficiency virus, antiretroviral therapy (ART), delayed initiation, treatment willingness, perception

## Abstract

**Introduction:**

Delayed antiretroviral therapy (ART) initiation is associated with poor HIV outcomes and a higher likelihood of HIV transmission.

**Methods:**

This cross-sectional study assessed the proportion of delayed ART initiation which was defined as initiating ART after 30 days of HIV diagnosis, and evaluated the pathways influencing ART initiation among adult PLWH in Changsha, China who were diagnosed between 2014 and 2022.

**Results:**

Of 518 participants, 37.8% delayed in initiating ART. Based on the theory of reasoned action (TRA), delayed initiation was indirectly associated with perceptions toward ART through the mediating pathway of patients’ treatment willingness, with treatment willingness significantly being the full mediator.

**Discussion:**

The findings may guide the development of interventions to improve timely uptake of ART in people who are newly diagnosed with HIV.

## 1. Introduction

Acquired immunodeficiency syndrome (AIDS) continues to be a global health crisis. Over 38 million people are living with HIV and 10 million do not have access to antiretroviral therapy (ART). There is no preventive vaccine or complete cure for HIV yet. However, people living with HIV (PLWH) can lead long and healthy lives by taking medicines that keep the virus undetectable, and people who maintain an undetectable viral load for at least six months are highly unlikely to transmit HIV through sex (1, 2). Since 2013, the Chinese Center for Disease Control and Prevention (CDC) has started a pilot program for early initiation of ART (3) and then rolled it out across the country in the subsequent years. ART coverage rate among PLWH in China was only 59.0% in 2014, and increased to 83.4% by 2018 (4). About 70% of HIV infected men who have sex with men intended to receive ART but about 60% were on ART in 2016 (5, 6). These findings suggested that there are significant gaps in ART initiation among PLWH in China. Delayed ART initiation can affect both treatment outcomes and the patient’s quality of life (7). The late onset of ART is strongly associated with lower immunologic response, poor virologic suppression, and a higher likelihood of developing related complications (8), and ultimately leads to treatment failure and even AIDS-related death (9). Studies have confirmed that timely initiation of ART can improve care engagement, shorten the duration of virologic suppression (10), and improve the health and life expectancy of HIV patients (9). Meanwhile, early initiation of ART significantly reduces HIV transmission (11). However, up to 30% of PLWH might delay their treatment both in developed and developing countries (12, 13, 14). The attitude and knowledge of clinicians (15), the interface of services between facilities, and limited resources have been reported as external factors affecting ART initiation (16, 17). Patients’ personal factors may also contribute to delayed initiation, such as demographic factors including low income, low education, and lack of household decision-making power (18), HIV-related characteristics (14), mental health (19, 20), and perceptions toward ART (21). Patients’ willingness to start treatment also influences the decision of ART initiation (22). However, no study has explored the pathway relationships and mechanisms of action among these factors. Identifying the possible pathways of influence will help with developing targeted interventions to promote timely ART initiation. We conducted a cross-sectional study in Changsha, China for assessing the perceptions toward ART and factors associated with delayed ART initiation among PLWH.

## 2. Methods

### 2.1. Study design and participants

This cross-sectional study was conducted among patients in the HIV clinic of a tertiary referral hospital in Changsha City, China. Patients who were aged 18 years or older and were diagnosed with HIV between 2014 and 2022 were invited to participate in the study. The sample size was calculated based on the precision of estimating the proportion of self-reported delay in ART initiation among participants. With an assumed 30% delay rate, a sample size of 323 or more participants is needed to have a 5% margin error with a 95% confidence level.

Each participant provided informed consent. The study was approved by the research ethics review committee of Xiangya School of Nursing, Central South University (NO. E2021134).

### 2.2. Data collection

A structured questionnaire was used to collect data during face-to-face interviews from October 2021 to June 2022. The questionnaire consisted of three parts: (1) socio-demographics including sex, age, place of residence, occupation, and sexual orientation; (2) HIV diagnosis and status including the year of HIV diagnosis, time of ART initiation, recent CD4 count, comorbidity; (3) perceptions toward ART and willingness to use ART.

Two investigators who had experience in working with PLWH conducted data collection. When patients were waiting to see a doctor in the HIV clinic, the clinic nurse referred them to meet the investigators. The investigator introduced the study and obtained a written consent from the patients who were interested in participating in the study and met the inclusion criteria. The investigators conducted a one-to-one interview with each participant in a private room and completed the questionnaire. They also verified key questionnaire information through cross-checking their electronic medical records including the dates of HIV diagnosis and ART initiation. If there were discrepancies between patient’s reported information and electronic records, the electronic records were used.

Delayed ART initiation: Delay ART initiation was defined if a participant started ART after 30 days of HIV diagnosis (23–25). This was based on self-report and was verified by checking patients’ electronic medical records.

Perceptions toward ART: The Health Belief Model-related ART perceptions scale was used to measure patients’ perceptions toward ART (21). This scale contains 8 domains including subjective norms, perceived severity, perceived susceptibility, perceived benefits, perceived barriers, perceptions on balance, self-efficacy, and cues to action. This scale has 26 items with a total score of 122, including 24 using a 5-point scale (from 1 = “extremely disagree” to 5 = “extremely agree”) and 2 using a yes or no option (1 = “yes,” 0 = “no”). A higher score represents high ART perception. In this study, this measure showed good internal consistency (Cronbach’s *α* = 0.830).

Treatment willingness: Participants were asked to rate their willingness to use ART on a scale from 1 to 10, with 1 representing the lowest willingness and 10 representing the highest willingness. The 10 points scoring format is simple to use and easier to understand for PLWH (26).

### 2.3. Statistical analysis

Double data entry was performed using the software Epidata 3.1. Descriptive and univariate analyses were performed using SPSS24.0. T-tests for continuous variables and chi-square (*χ*2) tests for categorical variables were used to compare two groups of delayed and timely ART initiation. A chi-square trend test was performed for the proportions of timely ART initiation over calendar years of HIV diagnosis.

**Figure 1:**
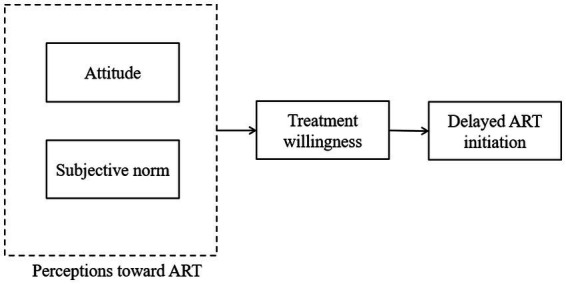
Hypothesized model for delayed initiation of ART based on the Theory of Reasoned Action. ART, antiretroviral therapy.

The theory of reasoned action (TRA) was used to guide the development of the research questions, hypotheses, and the path analysis model. TRA proposes that attitude and subjective norms indirectly influence the occurrence of behavior by acting on behavioral willingness within human action (27). We hypothesized that attitudes toward ART and subjective norms influence ART initiation among PLWH through the treatment willingness as a mediator (Figure 1). Path analysis was performed using Mplus8.3. We used six goodness of fit indices to assess the model, including *χ*^2^, the ratio of *χ*^2^ over the degree of freedom (*χ*^2^/df), comparative fit index (CFI), Tucker Lewis index (TLI), the root mean square error of approximation (RMSEA), and the standardized root mean square residual (SRMR). *χ*^2^ value with *p*>0.05, *χ*^2^/df between 1 and 3, RMSEA<0.07 (28), CFI and TLI values >0.95, and SRMR<0.08 (29) were considered acceptable. For the categorical outcome variable (delayed initiation or not), we used a Weighted Least Squares Mean Variance adjusted (WLSMV) parameter estimation algorithm which works best for the ordinal data in the Mplus (30). We calculated 95% confidence intervals (CIs) with 5,000 bootstrap samples for total effect, indirect effect, and direct effect.

## 3. Results

Of 536 patients who participated in the study, 18 (3.4%) did not complete the study procedures and were excluded, and 518 were included in the analysis. Age ranged from 18 to 76 years (mean, 32.2; standard deviation (SD), 10.3). About 96% were male. The average duration of HIV diagnosis was 4.2 (SD, 2.3) years. The average time from diagnosis to ART initiation was 4.1 (SD, 11.2) months. The average score of willingness to initiate ART after diagnosis was 8.1 (SD, 2.9), and the average score of perceptions toward ART was 90.4 (SD, 13.3).

**Figure 2:**
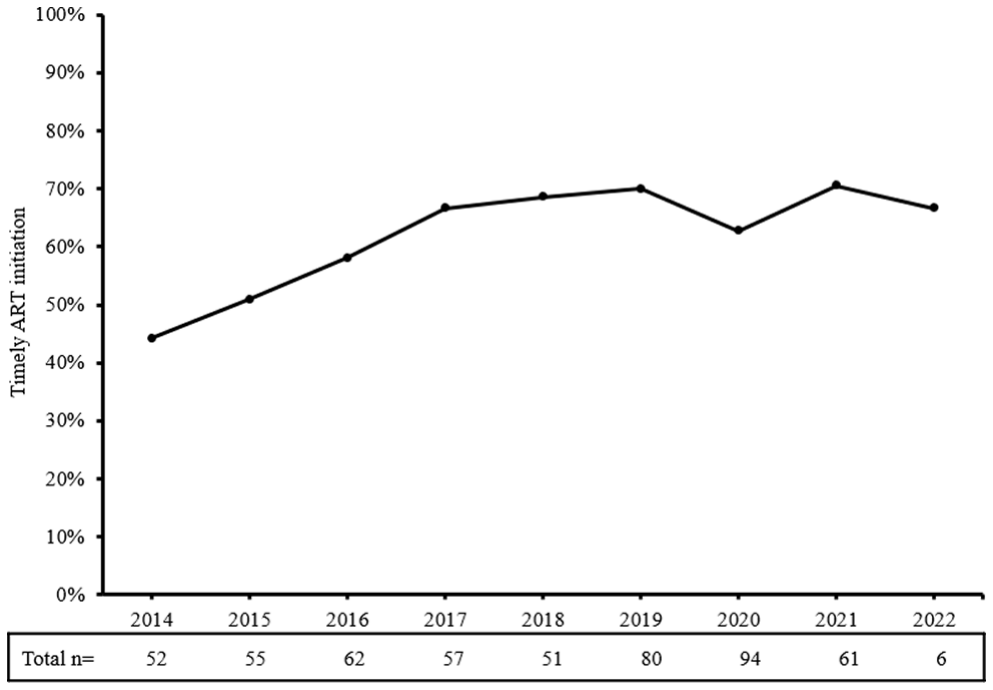
Proportions of timely initiation of ART among people who were newly diagnosed with HIV in the years 2014–2022. Timely initiation of ART has trended upward since 2014, although there was a small decline in 2020. ART, antiretroviral therapy.

About one-third (37.8%) of patients delayed in initiating ART treatment. Table 1 shows the characteristics of participants who delayed initiation of ART and those who timely initiated ART. Patients who delayed initiation were younger than those who had timely initiation. Year of HIV diagnosis was significantly associated with delayed ART initiation (*p*<0.01), and there was an increasing trend of ART timely initiation since 2014 (trend *χ*^2^ = 10.514, *p*<0.01, Figure 2). In addition, treatment willingness (*p*<0.001) and perceptions toward ART (*p*<0.001) were also significantly associated with delayed ART initiation.

**Table 1:** Demographic and health characteristics of participants by time of ART initiation.

Variable	Total (*n* = 518)		Delayed ART initiation (*n* = 196)		Timely ART initiation (*n* = 322)		*p*
	*M*	*SD*	*M*	*SD*	*M*	*SD*	
Age (years)^a^	32.2	10.3	31.0	9.5	33.0	10.8	0.03
Duration of HIV diagnosis(years)^a^	4.2	2.3	4.6	2.4	3.9	2.2	<0.01
Treatment willingness^a^	8.1	2.9	6.2	3.3	9.3	1.8	<0.001
Perceptions toward ART^a^	90.4	13.3	85.9	18.2	93.1	8.1	<0.001
	*N*	%	*n*	%	*n*	%	*p*
Sex
Male	498	96.1	191	97.5	307	95.3	0.23
Female	20	3.9	5	2.6	15	4.7	
Residency
Rural	264	49.0	102	52.0	162	50.3	0.70
Urban	254	51.0	94	48.0	160	49.7	
Education
High school or lower	97	23.1	32	16.3	65	20.2	0.36
Vocational school	182	37.3	79	40.3	103	32.0	
College or higher	239	39.6	85	43.4	154	47.8	
Employment status							
Unemployed	92	17.8	31	15.8	61	18.9	0.37
Employed	426	82.2	165	84.2	261	81.1	
Monthly household income (US$) (US dollars)
≤1,433	210	40.5	81	41.3	129	40.1	0.69
1,434–2,866	155	29.9	53	27.0	102	31.7	
2,867–4,299	61	11.8	24	12.2	37	11.5	
>4,299	92	17.8	38	19.4	54	16.8	
Sexual orientation
Heterosexual	122	23.6	37	18.9	85	26.4	0.25
Homosexual	274	52.9	112	57.1	162	50.3	
Bisexual	70	13.5	26	13.3	44	13.7	
Not sure	52	10.0	21	10.7	31	9.6	
Self-paid medications
No	249	48.1	91	46.4	158	49.1	0.56
Yes	269	51.9	105	53.6	164	50.9	
Comorbidities
No	444	6.8	162	82.7	282	87.6	0.10
Yes	35	85.7	13	6.6	22	6.8	
Unknown	39	7.5	21	10.7	18	5.6	
Alcohol use in the last 6 months
No	216	41.7	74	37.8	142	44.1	0.16
Yes	302	58.3	122	62.2	180	55.9	
Recent CD4 count (cells/mm^3^)
<200	31	6.0	15	7.7	16	5.0	0.16
200–349	103	19.9	31	15.8	72	22.4	0.23
>350	361	69.7	143	73.0	218	67.7	
Missing	23	4.4	7	3.6	16	5.0	

a Use *t*-tests for continuous variables; use chi-square tests for categorical variables.

M, mean; SD, standard deviation; ART, antiretroviral therapy; HIV, human immunodeficiency virus.

Age and year of HIV diagnosis were included in path analysis mode based on the univariate analysis and review (31). This model was tested and presented sufficient goodness of fit value, and the model fit indices included χ^2^ = 5.911 (*p* = 0.052), χ^2^/df = 2.955 (df = 2), CFI = 0.991, TLI =0.967, RMSEA = 0.061, and SRMR = 0.024. Table 2 shows that the standardized path coefficients (β) between delayed ART initiation and perceptions toward ART, treatment willingness, year of HIV diagnosis, and age are 0.022 (*p* = 0.72), −0.590 (*p* < 0.001), 0.242 (*p* < 0.001) and-0.173 (*p* < 0.01), respectively. Specifically, perceptions toward ART have a direct and positive association with treatment willingness (β = 0.598, *p* < 0.001).

**Table 2:** Path Model for parameter estimates of delayed ART initiation.

Pathway			*B*	*SE*	*β*	*p*
Perceptions toward ART	→	Treatment willingness	0.130^***^	0.038	0.598^***^	0.00
Perceptions toward ART	→	Delayed ART initiation	0.002	0.061	0.022	0.72
Treatment willingness	→	Delayed ART initiation	−0.223^***^	0.055	−0.590^***^	0.00
Year of HIV diagnosis	→	Delayed ART initiation	0.116^***^	0.054	0.242^***^	0.00
Age	→	Delayed ART initiation	−0.018^**^	0.061	−0.173^**^	0.00

***p < 0.001, **p < 0.01.

B, unstandardized coefficient; SE, standard error; *β*, standardized coefficient; ART, antiretroviral therapy; HIV, human immunodeficiency virus.

**Figure 3:**
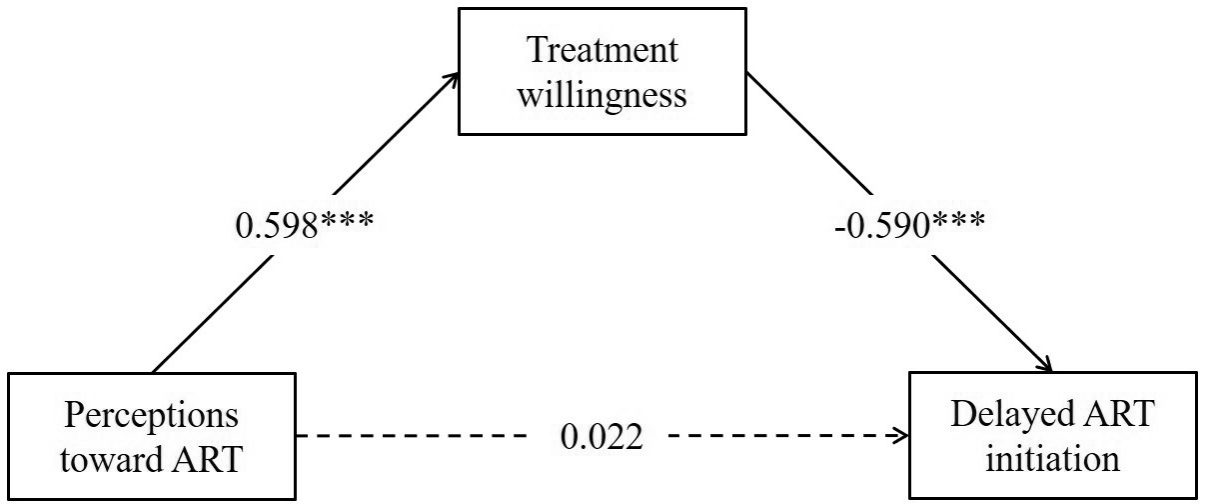
Model for perceptions toward ART and delayed ART initiation among people living with HIV in Changsha, China. The solid line indicates that the pathway is significant and the dashed line is insignificant. Treatment willingness plays a fully mediating role in the impact of perceptions toward ART on delayed ART initiation. ART, antiretroviral therapy. ***p < 0.001.

For the total mediated model (Figure 3 and Table 3), the total effect is-0.331 (*p* < 0.001, 95%CI: −0.458 to-0.191), the indirect effect is-0.353 (*p* < 0.001, 95%CI: −0.478 to-0.247), and the direct effect is 0.022 (*p* = 0.72, 95%CI: −0.143 to 0.171). The direct effect or the effect of perceptions toward ART on delayed ART initiation controlling for age and years of HIV diagnosis is non-statistically significant, therefore, treatment willingness plays a fully mediating role in the impact of perceptions toward ART on delayed ART initiation.

**Table 3:** Mediating effect for delayed ART initiation.

Effects	Two-tailed estimate	SE	Est./SE	p	Lower 95% CI	Upper 95% CI
Total effect	−0.331^***^	0.054	−6.077	0.00	−0.458	−0.191
Indirect effect	−0.353^***^	0.046	−7.687	0.00	−0.478	−0.247
Direct effect	0.022	0.061	0.359	0.72	−0.143	0.171

***p < 0.001.

SE, standard error; Est./SE, Two-tailed estimate/Standard error; 95% CI, 95% confidence interval.

## 4. Discussion

This study showed a 37.8% proportion of delayed ART initiation among PLWH in Changsha, China during 2014–2022. It is significantly lower than the national average between the year 2011–2014 (76.6%) (25) and lower than the average rate during 2010–2015 in San Francisco, United States (62.0%) (32) and it is close to the rates reported in some studies in low-and middle-income countries, such as South Africa (33). Due to the changing definition of delayed ART initiation (8), caution should be taken when comparing the rates across regions and countries. While we used the definition in the current version of Chinese national ART guidelines, other studies may use different definitions, such as a specific CD4 count or a different duration from HIV diagnosis to ART initiation (24). This variation in definition may contribute to difference in the reported rates of delayed ART initiation so cautions should be taken when making direct comparison with other studies.

The overall trend of timely initiation rates increased over calendar years, and this is consistent with another study in China (34). It may be due to the continuous optimization of treatment policies and improvement of the HIV treatment environment (35). The decline in 2020 may be related to COVID-19 outbreak (36). ART interruption in 2022 has also been found in other regions such as South Africa (37). Measures taken to slow the transmission of COVID-19 and the additional strain placed on health systems have disrupted HIV treatment services (38). The greatest disruptions occurred in the first half of 2020 when many countries were in their first emergency embargo (37). Having access to timely ART was a concern in HIV care during the COVID-19 pandemic (39). In addition, morbidity and mortality associated with COVID-19, fear of transmission, and government response policies might lead to a reduction in ART initiation, especially for the vulnerable population among PLWH (37, 40).

This study demonstrated that the relationship between perceptions toward ART and delayed ART initiation was fully mediated by treatment willingness. It is consistent with findings from both qualitative and quantitative studies. A consolidation of qualitative evidence covering high-income countries, and low-and middle-income countries showed that perceptions toward ART were associated with delayed ART initiation (22, 41). Two cohort studies also demonstrated this association (42, 43). Poloko and colleagues found that individual treatment willingness also influenced ART initiation (44). However, unlike previous studies, our study found no direct impact of perceptions toward ART on delayed initiation, but rather through the mediation of treatment willingness. We need to be aware that there is a know-do gap between perceptions toward ART and initiation behavior (45). In practice, interventions that simply enhance ART perceptions may not directly help patients initiate ART as rapidly as possible; we still need to be mindful of patients’ treatment willingness and design targeted interventions to ensure that all patients are not left behind.

Our study pointed to a relationship between younger age and delayed ART initiation. Our findings were similar to a Swiss prospective cohort study, although delayed ART initiation of that study was defined by CD4 counts (46). A Canadian study that also used days to determine delayed initiation as we did was also consistent with our findings (14). However, unlike the study by Hassan et al. Age was not significantly associated with delayed initiation in their study. A historical cohort study contradicted us and concluded that older age was associated with delayed ART initiation (47). Such a difference may be due to the different regions and study periods of our study.

In addition, we found that economic-related components, including employment status, monthly household income, and self-paid medications were none significant. In general, researchers believe that patients with higher economic status are more likely to start ART early (14, 48). Yet, one study even found that monetary incentives continued to fail to facilitate ART initiation as soon as possible (49). These differences may be influenced by culture and policy in different study contexts. This also suggests that we can also try to explore new non-financial interventions to help patients initiate ART as soon as possible.

Our study validated the TRA model. First, our study further validated the theory by confirming the mediating role of treatment willingness. Second, we broadened the application of TRA in HIV care scenarios. In previous studies, TRA has been used mainly for medication adherence (50, 51), HIV self-testing (52), and condom use (53). To our knowledge, this is the first study of innovatively applying the TRA model for explaining ART initiation among PLWH, and study findings may serve as a scientific basis for developing interventions on ART treatment.

The study has limitations. First, this study does not provide causal inferences between perceptions toward ART and initiation of ART due to the nature of cross-sectional study design. Prospective cohort studies and intervention clinical trials are needed to assess the causal relationship. Second, data on perceptions and initiation of ART are based on self-report, and therefore, recall bias may exist. As patients were newly diagnosed with HIV between 2014 and 2022, most had been in treatment for a long time when they participated in this survey, and this may have influenced the findings on ART perceptions and reported dates on HIV diagnosis and ART initiation. We guided study participants through recalling their situation at the time of HIV diagnosis and incorporated prompts in the questionnaire to assist with memory recall. The information on the dates of HIV diagnosis and ART initiation was verified by checking patients’ electronic medical records to minimize the recall bias. However, recall bias is still possible. Third, although the number of participants exceeded the required sample size, only 3.9% of the participants were female. The low proportion of female participants may limit the generalizability of our study findings. Forth, delayed ART initiation was defined as initiating ART after 30 days of HIV diagnosis in this study regardless of patients’ disease status, but the recommendations for the time of initiating ART in both international and Chinese ART guidelines have been changing over time. This definition we used in this study may or may not cause bias in assessing the relationship between ART perception and initiation. Finally, this study was conducted in one city and the same participants could not represent all PLWH in China. Further research is warranted to replicate the study findings in other parts of China.

## 5. Conclusion

In conclusion, this study found a high proportion of delayed ART initiation among PLWH in Changsha, China and a fully mediating role of treatment willingness between perceptions toward ART and delayed ART initiation. The findings may guide the development of interventions for reducing delayed initiation.

## Data availability statement

The raw data supporting the conclusions of this article will be made available by the authors, without undue reservation.

## Ethics statement

The studies involving human participants were reviewed and approved by the research ethics review committee of Xiangya School of Nursing, Central South University. Written informed consent for participation was not required for this study in accordance with the national legislation and the institutional requirements.

## Author contributions

YZ: design of methodology (lead), investigation (equal), data curation (lead), formal analysis (lead), visualization (lead), software (lead), original draft (lead), and review and editing (equal). YL: investigation (equal), data curation (supporting), and validity (lead). XX: conceptualization (equal) and design of methodology (supporting). H-ZQ: review and editing (equal). HW: supervision (lead), project administration (lead), funding acquisition (lead), conceptualization (equal), and review and editing (equal). All authors contributed to the article and approved the submitted version.

## Funding

This research was supported by the National Natural Science Foundation of China (No. 82273746) and the Provincial Natural Science Foundation of Hunan grant (No. 2022JJ30769). This research was also supported by the Hunan Provincial Innovation Foundation for Postgraduate (No. CX20220337) and the Fundamental Research Funds for the Central Universities of Central South University (No. 1053320213461).

## Conflict of interest

The authors declare that the research was conducted in the absence of any commercial or financial relationships that could be construed as a potential conflict of interest.

## Publisher’s note

All claims expressed in this article are solely those of the authors and do not necessarily represent those of their affiliated organizations, or those of the publisher, the editors and the reviewers. Any product that may be evaluated in this article, or claim that may be made by its manufacturer, is not guaranteed or endorsed by the publisher.
